# Inverse stochastic resonance induced by ion channel noise

**DOI:** 10.1186/1471-2202-13-S1-P181

**Published:** 2012-07-16

**Authors:** Muhammet Uzuntarla, John R Cressman, Mahmut Ozer, Ernest Barreto

**Affiliations:** 1 Departments of Biomedical Engineering, University of Karaelmas, 67100 Zonguldak, Turkey; 2 School of Physics, Astronomy, and Computational Sciences, and The Krasnow Institute for Advanced Study, George Mason University, Fairfax, VA 22030, USA; 3 Department of Electrical and Electronics Engineering, University of Karaelmas, 67100 Zonguldak, Turkey

## 

Recent work has considered the inhibitory effects of noise on neuronal activity, particularly on rhythmic firing. For example, Paydarfar et al. [1] studied the influence of noise on neuronal pacemakers in an *in vitro* preparation of the squid giant axon, and found that small noisy currents induce an on-off switching behavior between two nearby regimes: repetitive firing and quiescence. They also showed that the timings of on-off switching of the pacemaker depend on the intensity and spectral properties of noisy current. Tuckwell et al. [2,3] further investigated the inhibitory effect of noise in a single Hodgkin-Huxley neuron. These authors show that in a model neuron subject to stochastic external additive noise, the average firing rate exhibits a minimum as the noise amplitude is varied. The authors called this phenomenon Inverse Stochastic Resonance (ISR), in contrast to the well-known phenomenon of stochastic resonance.

**Figure 1:**
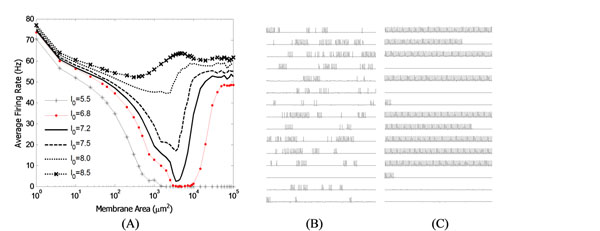
(A) Inverse stochastic resonance: average firing rate versus membrane area for different values of constant input current I_0_. Effective channel noise intensity is smaller for larger membrane area. Right: sample traces from random initial conditions used in calculating (A), drawn from (B) area=750μm^2^ and (C) area=30000μμm^2^, with I_0_=6.8 μμcm^-2^.

In these modelling studies [2,3], noise was incorporated by adding an external noisy current. Here, we consider the ISR phenomenon in the Hodgkin-Huxley neuron with a more biophysically realistic model of noise: that resulting from the stochastic nature of voltage-gated ion channels embedded in neuronal membranes. The intensity of channel noise is related to the total number of channels for fixed channel density, i.e. when the number of ion channels (or the cell size) is small, the stochasticity of channels imparts strong noise intensity to the neuron’s dynamics. Our results show that the ISR phenomenon is also present in the case of ion channel noise. We clarify the mechanism that underlies ISR and show that the most surprising feature – the increase in average firing rate as the noise decreases (membrane area increases) – is a consequence of the dynamical structure of the model and the averaging procedure (Figure 1). We also discuss the relative contributions of the different channel subunits to transitions from the spiking to the rest state, and vice versa, in the noisy case.
